# Supplementary material for: From sequence to enzyme mechanism using multi-label machine learning
Source: BMC Bioinformatics. 2014 May 19;15:150. doi: 10.1186/1471-2105-15-150 (PMC4229970; doi:10.1186/1471-2105-15-150)
Supplement: Additional file 2 — Java code of ml2db. Additional file ml2db_code.tar.gz contains the Java source code to run the multi-label machine learning experiments and save the results to database. The code’s Javadoc is included. [file 1471-2105-15-150-S2.zip › additional file 2/ml2db/ml2db/doc/mulan/evaluation/Evaluator.html]

Evaluator


JavaScript is disabled on your browser.


- Overview
- Package
- Class
- Use
- Tree
- Deprecated
- Index
- Help

- Prev Class
- Next Class

- Frames
- No Frames

- All Classes

- Summary:
- Nested |
- Field |
- Constr |
- Method

- Detail:
- Field |
- Constr |
- Method


mulan.evaluation

## Class Evaluator

- java.lang.Object
- - mulan.evaluation.Evaluator

- ---

    

  ```
  public class Evaluator
  extends java.lang.Object
  ```

  Evaluator - responsible for generating evaluation data

  Version:
  :   2011.09.06

  Author:
  :   rofr, Grigorios Tsoumakas

- - ### Constructor Summary

    Constructors

    Constructor and Description

    `Evaluator()`
  - ### Method Summary

    Methods

    | Modifier and Type | Method and Description |
    | --- | --- |
    | `void` | `checkData(MultiLabelInstances data)` |
    | `void` | `checkLearner(MultiLabelLearner learner)` |
    | `MultipleEvaluation` | `crossValidate(MultiLabelLearner learner, MultiLabelInstances data, int someFolds)` Evaluates a `MultiLabelLearner` via cross-validation on given data set with defined number of folds and seed. |
    | `MultipleEvaluation` | `crossValidate(MultiLabelLearner learner, MultiLabelInstances data, java.util.List<Measure> measures, int someFolds)` Evaluates a `MultiLabelLearner` via cross-validation on given data set using given evaluation measures with defined number of folds and seed. |
    | `Evaluation` | `evaluate(MultiLabelLearner learner, MultiLabelInstances data)` Evaluates a `MultiLabelLearner` on given test data set. |
    | `Evaluation` | `evaluate(MultiLabelLearner learner, MultiLabelInstances data, java.util.List<Measure> measures)` Evaluates a `MultiLabelLearner` on given test data set using specified evaluation measures |
    | `void` | `setSeed(int aSeed)` Sets the seed for reproduction of cross-validation results |

    - ### Methods inherited from class java.lang.Object

      `equals, getClass, hashCode, notify, notifyAll, toString, wait, wait, wait`

- - ### Constructor Detail


    - #### Evaluator

      ```
      public Evaluator()
      ```
  - ### Method Detail


    - #### setSeed

      ```
      public void setSeed(int aSeed)
      ```

      Sets the seed for reproduction of cross-validation results

      Parameters:
      :   `aSeed` - seed for reproduction of cross-validation results


    - #### evaluate

      ```
      public Evaluation evaluate(MultiLabelLearner learner,
                        MultiLabelInstances data,
                        java.util.List<Measure> measures)
                          throws java.lang.IllegalArgumentException,
                                 java.lang.Exception
      ```

      Evaluates a `MultiLabelLearner` on given test data set using specified evaluation measures

      Parameters:
      :   `learner` - the learner to be evaluated via cross-validation
      :   `data` - the data set for cross-validation
      :   `measures` - the evaluation measures to compute

      Returns:
      :   an Evaluation object

      Throws:
      :   `java.lang.IllegalArgumentException` - if an input parameter is null
      :   `java.lang.Exception`


    - #### checkLearner

      ```
      public void checkLearner(MultiLabelLearner learner)
      ```


    - #### checkData

      ```
      public void checkData(MultiLabelInstances data)
      ```


    - #### evaluate

      ```
      public Evaluation evaluate(MultiLabelLearner learner,
                        MultiLabelInstances data)
                          throws java.lang.IllegalArgumentException,
                                 java.lang.Exception
      ```

      Evaluates a `MultiLabelLearner` on given test data set.

      Parameters:
      :   `learner` - the learner to be evaluated
      :   `data` - the data set for evaluation

      Returns:
      :   the evaluation result

      Throws:
      :   `java.lang.IllegalArgumentException` - if either of input parameters is null.
      :   `java.lang.Exception`


    - #### crossValidate

      ```
      public MultipleEvaluation crossValidate(MultiLabelLearner learner,
                                     MultiLabelInstances data,
                                     int someFolds)
      ```

      Evaluates a `MultiLabelLearner` via cross-validation on given data set with defined number of folds and seed.

      Parameters:
      :   `learner` - the learner to be evaluated via cross-validation
      :   `data` - the multi-label data set for cross-validation
      :   `someFolds` -

      Returns:
      :   a `MultipleEvaluation` object holding the results


    - #### crossValidate

      ```
      public MultipleEvaluation crossValidate(MultiLabelLearner learner,
                                     MultiLabelInstances data,
                                     java.util.List<Measure> measures,
                                     int someFolds)
      ```

      Evaluates a `MultiLabelLearner` via cross-validation on given data set using given evaluation measures with defined number of folds and seed.

      Parameters:
      :   `learner` - the learner to be evaluated via cross-validation
      :   `data` - the multi-label data set for cross-validation
      :   `measures` - the evaluation measures to compute
      :   `someFolds` -

      Returns:
      :   a `MultipleEvaluation` object holding the results


- Overview
- Package
- Class
- Use
- Tree
- Deprecated
- Index
- Help

- Prev Class
- Next Class

- Frames
- No Frames

- All Classes

- Summary:
- Nested |
- Field |
- Constr |
- Method

- Detail:
- Field |
- Constr |
- Method
